# No Association Between HIV-1 Subtype and Primary Resistance Mutations with CD4 Reconstitution During Effective Antiretroviral Treatment: An Observational, Cohort Study

**DOI:** 10.3390/ijms26041410

**Published:** 2025-02-07

**Authors:** Andrzej Załęski, Agnieszka Lembas, Tomasz Dyda, Joanna Osińska, Joanna Jabłońska, Justyna Stempkowska-Rejek, Justyna Orzechowska, Alicja Wiercińska-Drapało

**Affiliations:** 1 Hospital for Infectious Diseases in Warsaw, 01-201 Warsaw, Poland; andrzejzaleski84@wp.pl (A.Z.);; 2 Department of Infectious Diseases, Tropical Diseases and Hepatology, Medical University of Warsaw, 02-091 Warsaw, Poland; 3 Molecular Diagnostics Laboratory, Hospital for Infectious Diseases in Warsaw, 01-201 Warsaw, Poland; 4 Infectious Diseases Clinical Ward in Ostróda, Department of Family Medicine and Infectious Diseases, University of Warmia and Mazury in Olsztyn, 10-719 Olsztyn, Poland; 5 Department of Infectious Diseases and Hepatology, Medical University of Lublin, 20-059 Lublin, Poland; 6 Medical Center in Łańcut, Clinical Department of Infectious Diseases, College of Medical Sciences, University of Rzeszów, 35-959 Rzeszów, Poland

**Keywords:** HIV-1, HIV subtype, DRMs, immune reconstitution, CD4+ T lymphocytes, mutations, drug resistance

## Abstract

Some people with Human Immunodeficiency Virus (HIV) on effective antiretroviral therapy have persistent low lymphocyte CD4 counts and remain at an increased risk of Acquired Immunodeficiency Syndrome (AIDS). We investigated whether primary drug resistance mutations (DRMs) and HIV-1 subtype could be related to immunologic reconstitution in these people. In a multicenter, observational cohort study among treatment-naïve patients, we analyzed HIV-1 subtype, primary drug resistance mutations, CD4 counts, and CD4:CD8 ratios during effective antiretroviral therapy. We compared these variables between patients with different HIV subtypes and between those with or without drug-resistance mutations up to 48 weeks post-baseline. In 156 patients, CD4 count normalization (≥500 cells/µL) was observed in 39% of patients, while CD4:CD8 ratio ≥ 1 in 27% after treatment implementation. HIV-1 subtype B was present in 75% of the patients and subtype A in 22%. Primary resistance mutations were found in 57% of the individuals. The percentage of immunological nonrespondents did not differ significantly between those with different HIV subtypes or between those with or without primary resistance mutations (*p* > 0.05). In conclusion, there was no significant coincidence between the HIV subtype and primary drug resistance mutations with immunological reconstitution in patients receiving effective antiretroviral therapy.

## 1. Introduction

Untreated Human Immunodeficiency Virus (HIV) infection causes progressive and continuous impairment of the immune system, which results in the development of opportunistic infections and neoplasms. Current studies suggest that people with HIV (PWH) who are receiving effective antiretroviral therapy (ART) have similar life expectancies as non-HIV-infected individuals, despite the higher incidence of comorbidities, as effective ART can prevent the loss of CD4 T cells and, sometimes, help to restore them [1]. However, some patients present with persistently lower lymphocyte CD4 T cell counts and CD4:CD8 ratios despite virologically effective ART. This subgroup of PWH, referred to as immunological non-responders (INRs), remains at an increased risk of mortality due to progression to acquired immunodeficiency syndrome (AIDS) and other non-AIDS events [2,3].

The mechanisms of failed immunological reconstitution in the INRs are complex and multifactorial, with risk factors including older age, male sex, a low CD4 T cell count, and a low CD4:CD8 ratio at baseline [4,5]. Persistent inflammatory activation due to HIV infection and coinfections can also cause inadequate immune reconstitution, and the molecular basis of this phenomenon is the subject of many studies [6,7,8,9]. Despite ART, HIV may replicate continuously in the reservoirs, which can also cause chronic immune stimulation and inflammation and, thus, inadequate immune reconstitution [10,11,12]. On the other hand, the repopulation of CD4 cells after treatment starts may lead to immune reconstitution inflammatory syndrome (IRIS) [13]. Excessive immune response to pathogens, invigorated by functional impairments among regulatory T cells, may further induce proinflammatory activation, a possible risk factor for CD4 depletion [6,14,15].

At the molecular level, immune reconstitution differences among PWH may result not only from host polymorphisms and the duration of untreated infection but also from viral genetics. The differential molecular characteristics of HIV subtypes may influence HIV transmission, treatment outcomes, and disease progression. Some HIV subtypes and clusters are associated with a lower likelihood of achieving immune reconstitution or with a worse course of infection despite effective ART [16,17]. Some studies found that non-subtype A infections are associated with a higher risk of developing AIDS, but other studies reported conflicting results [17,18,19,20,21].

It has been demonstrated that in a population of PWH exposed to ART, where the B subtype dominates, the prevalence of drug resistance mutations (DRMs) is higher than in ART-naïve regions [22,23,24]. The genetic barrier, defined as the number of mutations required to overcome drug selective pressure, is an important factor contributing to the development of HIV drug resistance [25]. Thus, DRMs play a crucial role in the immune reconstitution process by modulating ART effectiveness. Primary, i.e., transmitted, mutations occur in ART-naive PWH. These people start treatment with a lower genetic barrier, a greater risk of virological failure, and a greater risk of developing resistance to other drugs, which may lead to insufficient immune reconstitution and progression to AIDS. Acquired DRMs, in turn, appear in PWH on inadequate ART and can be then transmitted to other people. In high-income countries, the prevalence of HIV infections with acquired DRMs is decreasing, but the prevalence of transmitted resistance is increasing. The prevalence of major primary HIV DRMs reported worldwide ranges from 5 to 15%, whereas the prevalence of major acquired DRMs in people with treatment failure reaches 50 to 70% [26,27,28,29,30].

Outcomes are related to subtype differences and resistance mutations, highlighting the importance of analyzing the features of HIV subtypes in the occurrence of DRMs, particularly when drug-resistant strains emerge [31,32,33]. Because the reasons for failed immune reconstitution remain unclear, we analyzed whether viral features, such as HIV subtype and the presence of primary DRMs, are associated with the reconstitution of CD4 cells during effective ART.

## 2. Results

### 2.1. Study Population

We recruited 156 patients for this study. Among them, 136 patients had regular CD4 cell counts and CD4:CD8 ratios, assessed up to 24 weeks after ART initiation, and 109 individuals had CD4 cell counts and CD4:CD8 ratios assessed up to 36 to 48 weeks post-baseline. Among the 136 patients assessed for up to 24 weeks, 24 individuals had either a CD4 cell count ≥500 cells/µL or a CD4:CD8 ratio ≥ 1 before ART initiation. Among the remaining 112 patients, 46 individuals (41.07%) achieved CD4 cell count normalization, and 24 patients (21.43%) achieved CD4:CD8 ratio normalization. The CD4 T cell count and CD4:CD8 T cell ratio normalization rates did not vary significantly between patients with 24- and 36- to 48-week observation periods (*p* = 0.222 and *p* = 0.076, respectively). Therefore, we performed further analyses on the group of 109 patients who were being observed for 36 to 48 weeks post-treatment. At the beginning of ART, 19 of these 109 patients (17.43%) had either a CD4 cell count ≥500 cells/µL or a CD4:CD8 ratio ≥ 1.

HIV type 1 (HIV-1) subtype A was present in 22% of the cases, and subtype B accounted for 75% of infections. The three patients with variant CRF02_AG and subtype C variants were excluded from the subtype analysis. DRMs were observed in 57% of the patients; the most frequent mutations (46%) were associated with protease inhibitors (PIs). We detected DRMs to nucleoside reverse transcriptase inhibitors (NRTIs): M184I, T215V/S, T69N, V118I, T39N, and A62V; DRMs to nonnucleoside reverse transcriptase inhibitors (NNRTIs): E138A, V106I, V179D, K101Q0; and DRMs to PIs: L10I/V/F, A71T/V, and Q58E. Besides the A71T mutation, which was observed more frequently in the B subtype (*p* = 0.037), no significant differences between subtypes and the presence of specific DRMs were detected (*p* > 0.05).

In all analyzed patients, tailored ART was introduced and continued uninterruptedly: 52% of individuals received integrase strand transfer inhibitor-based (InSTI) therapy, and 24% received either PI- or NNRTI-based therapy. There were no statistically significant differences in the ΔCD4 count or the ΔCD4:CD8 ratio by treatment type (*p* = 0.989 and *p* = 0.996, respectively).

**Figure 1:**
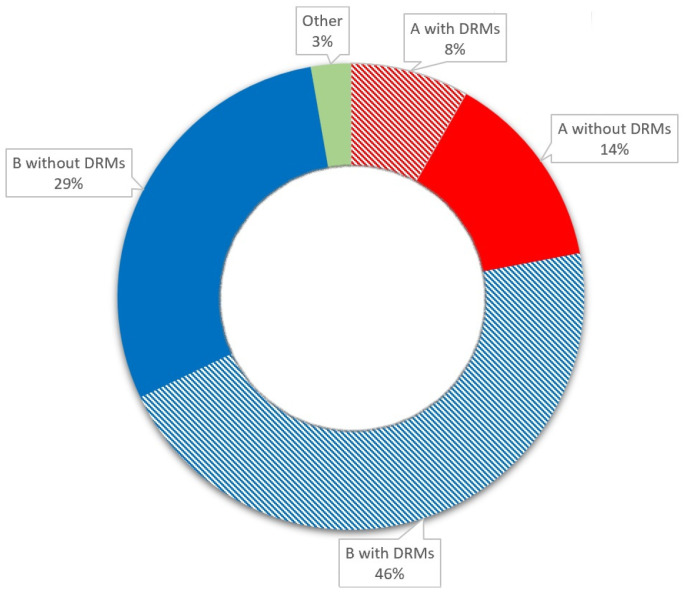
Baseline patient characteristics.

Up to 48 weeks after ART initiation, CD4 count normalization was observed in 39% of patients, and CD4:CD8 ratio normalization was observed in 27% of patients. The baseline patient characteristics are presented in Table 1 and Figure 1.

### 2.2. HIV Subtype-Dependent Restoration

**Figure 2:**
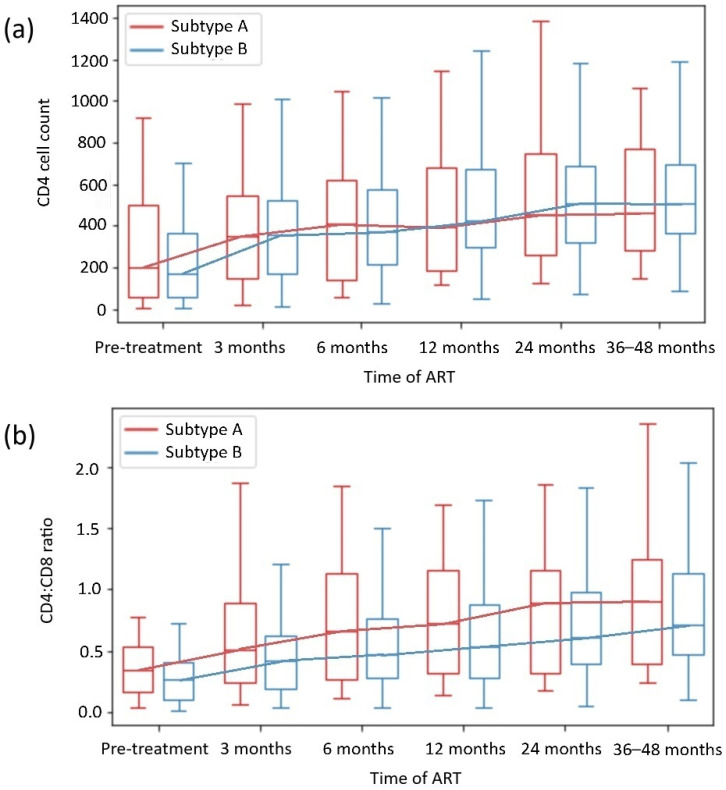
CD4+ T-cell count (**a**) and CD4+ T cell:CD8+ T cell ratio (**b**) recovery depending on HIV subtype.

The analysis of the CD4 count and CD4:CD8 ratio during the observation period by HIV subtype and the presence of HIV DRMs is shown in Figure 2 and Table 2 and Table 3. The analysis revealed no significant differences between the HIV subtypes A and B or groups of INRs.

In addition, the subgroup analysis revealed no significant differences in the CD4 count or CD4:CD8 ratio normalization in most subgroups with or without DRMs. Only patients with HIV-1 subtype B with DRMs achieved higher CD4 counts than those with HIV-1 subtype B without DRMs; likewise, they achieved higher ΔCD4:CD8 ratios than those with HIV-1 subtype A with DRMs (*p* = 0.013 and *p* = 0.047, respectively).

We also analyzed whether known risk factors for being an INR (male sex, older age, a low CD4 count, a low CD4:CD8 ratio at diagnosis, and the AIDS stage) were different between subtype subgroups, which could influence the results. Apart from the male sex, which was more common in the subtype B subgroup (*p* = 0.04), no significant differences were detected in the subgroup population (Table 1).

### 2.3. DRM-Dependent Restoration

**Figure 3:**
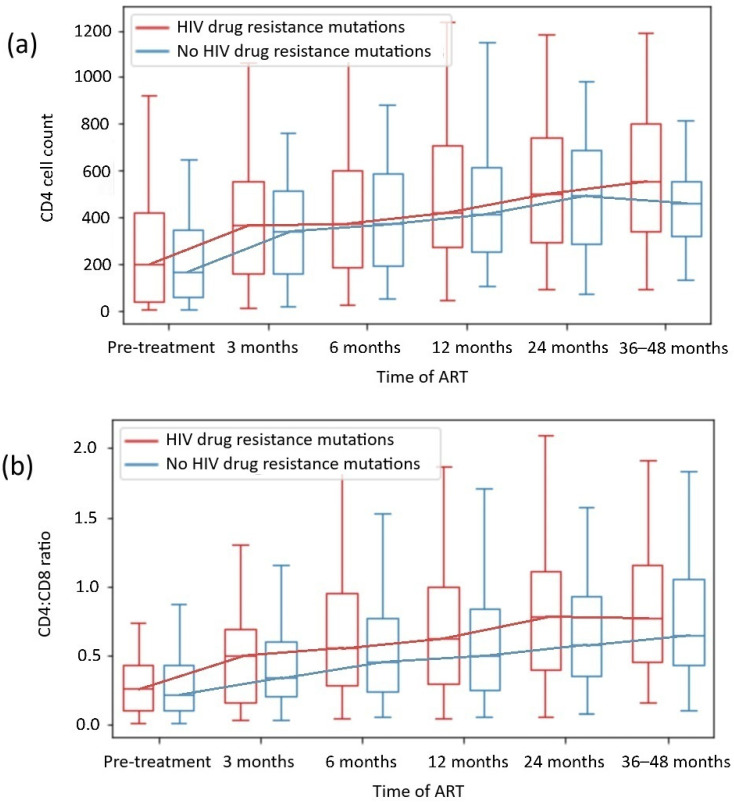
CD4 + T cell count (**a**) and CD4+CD8+ T cell ratio recovery (**b**) depending on HIV drug resistance mutations.

We did not observe that the presence of DRMs, including different drug class mutations, was associated with worse immunological restoration in any of the subgroups, including the INR subgroup. The analysis of the CD4 count and the CD4:CD8 ratio during the observation period among patients with and without HIV DRMs is presented in Figure 3, and the analysis of the CD4 count and the CD4:CD8 ratio by specific drug class mutation is shown in Table 4.

The analysis of known risk factors for INRs in patient subgroups with and without DRMs revealed that a younger median age and male sex were more common in the subgroup with DRMs (*p* = 0.003 and *p* = 0.001, respectively). No other significant differences among subgroups were detected (Table 1).

## 3. Discussion

### 3.1. Time of Observation of CD4 Restoration

Immune reconstitution in PWH is a process of rebuilding the immune system after the introduction of ART. The Centers for Disease Control and Prevention (CDC) considers a CD4 cell count ≥ 500 cells/µL and a CD4:CD8 ratio ≥ 1 in the normal range; achieving these levels is one of the goals of HIV treatment [34]. Reports suggest that the CD4:CD8 ratio may demonstrate immune dysfunction in patients with well-controlled HIV infection better than the CD4 cell count alone [35]. The results of these studies suggest that longer ART is associated with higher CD4:CD8 ratio normalization rates. Multiple trials have confirmed that patients achieve CD4:CD8 ratio normalization with a median duration of ART of 4 years [5,36]. The highest rates of CD4 cell count and CD4:CD8 ratio increase were observed during the first 2 years of effective ART [4,5]. Nevertheless, studies have shown that even long-term ART rarely leads to the normalization of the CD4 count and CD4:CD8 ratio, which is observed in only 10 to 45% of successfully treated patients [3,36,37,38].

In our analysis, within 48 weeks of observation, CD4 T cell count normalization was observed in 39% of patients, and CD4:CD8 ratio normalization was observed in 27% of patients. The CD4 cell count and CD4:CD8 ratio normalization rates did not vary significantly between patients in the 24-week observation period and those in the 36- to 48-week observation period (*p* = 0.222 and *p* = 0.076, respectively). On that basis, 24 to 48 months of observation in our study were representative for evaluating patients’ degree of immune reconstitution, which aligns with the outcomes of other clinical trials, with a significant increase in CD4 count during the first 2 years of effective ART—Figure 2 and Figure 3 [4,5,36,37,38].

### 3.2. HIV Subtype-Dependent Analysis

#### 3.2.1. HIV Subtype Epidemiology

HIV-1 is responsible for approximately 95% of all HIV infections worldwide, and it is also the dominant type in Europe, including in Poland. Within the most widespread HIV-1 group M, subtype C accounted for almost half (47%) of the infections, B accounted for 12%, and A accounted for 10% worldwide. HIV subtypes can combine to form hybrids such as circulating or unique recombinant forms (CRF or URF). Both CRFs, CRF02_AG and CRF01_AE, account for 13% of HIV cases. Subtype B predominates in both Americas, western Europe (75%), and Oceania, whereas subtype A remains the most prevalent strain (>50%) in Russia, former Soviet Union countries, and East Africa [39].

The different subtypes predominate in certain geographic areas, but their distribution is becoming increasingly heterogeneous as the HIV pandemic progresses. In the past decades, the increasing prevalence of non-B subtypes in Europe has been reported, and non-B subtypes have become endemic in the European population, not only in migrants from former Soviet Union countries [32]. In our cohort, 75% of the patients were Polish and 20.5% were Ukrainian. We detected subtype B in 75% of the patients and subtype A in 22%. The increasing number of cases of HIV infection with subtype A in Poland result from war migration, mainly from Ukraine, where, due to generalized HIV epidemics, more patients are involved in heterosexual transmission [40]. Continuous changes in HIV epidemiology make up-to-date surveillance for DRMs essential for all HIV subtypes and their recombinants.

#### 3.2.2. HIV Subtype Differences

Because of HIV-1’s high genetic variability, there is no standard wild-type strain. For drug resistance studies, mutations are defined as amino acid differences from one of several wild-type reference sequences. The HXB2, NL43, and subtype B consensus reference sequences are most commonly used for laboratory virus isolates. DRMs are generally found across all subtypes, but the use of the subtype B consensus has prevented the prevalence and significance of DRMs in non-B subtypes, including recombinants, from being fully explored [26,27]. Some studies indicate no major differences in DRM distribution between subtypes [29], while others do [41].

Some data also show that the HIV-1 subtype does not affect patients’ response to treatment [42], but differential pharmacogenomics of HIV-1 subtypes and their interactions with human hosts may influence the host’s ability to respond to ART, resulting in insufficient immune reconstitution and disease progression to AIDS. This concern has been demonstrated in cases of group O strains of HIV-1 and HIV-2, which present intrinsic resistance to NNRTIs, and by CRF01_AE, which is resistant to fostemsavir [43,44]. The likelihood of AIDS development is greater in patients infected with a non-A subtype, but the data from the studies indicating this likelihood are inconsistent [16,17,20,45]. Furthermore, different courses of HIV infection and progression to AIDS in PWH who were successfully treated with ART also have been demonstrated [16].

Results from studies show that in the population of PWH-exposed ART, where the B subtype dominates, the prevalence of drug resistance mutations (DRMs) is higher than in ART-naïve regions [22,23,24]. In our study, besides the A71T mutation, which was observed more frequently in the B subtype (*p* = 0.037), no significant differences between subtypes and the presence of specific DRMs were detected (*p* > 0.05). We have found that patients with subtype B infections were males more frequently (*p* < 0.05); moreover, younger age and male sex were also associated with infections with DRMs strains (*p* < 0.05). These findings are consistent with epidemiological data concerning populations with ART exposure [31,32,33,39,40].

#### 3.2.3. Possible HIV Subtype Influence on Immune Reconstitution

Immune reconstitution is influenced primarily by the implementation of ART, which should be introduced as soon as possible, preferably during primary HIV infection. Long-term and untreated infection causes irreversible damage to the immune system and, therefore, a poor immunological response to ART [3,4,5]. Moreover, immune reconstitution disparities in virologically suppressed PWH may result not only from host polymorphisms and the duration of untreated infection but also from viral genetics: some HIV subtypes and clusters are associated with a poorer probability of achieving immune reconstitution or a worse course of infection, also despite effective ART [16,17].

Our analysis of HIV-1 subtype-dependent CD4 cell and CD4:CD8 ratio reconstitution revealed no differences in immune restoration in virologically suppressed patients on continuous ART, although male sex was more common in the subtype B subgroup. The analysis of DRM-subtype subgroups also revealed no significant differences in the CD4 cell count or the CD4:CD8 ratio normalization across groups. Interestingly, within the B subtype, patients with DRMs had higher CD4 counts than those without DRMs (*p* = 0.013). When patients with the A and B subtypes with DRMs were compared, those with B subtypes achieved a greater ΔCD4:CD8 (*p* = 0.047). In regions where the HIV-1 B subtype dominates, and DRMs are more frequent, the availability of ART is also higher, and the results of HIV management are better [31,32,33]. Thus, not only ART implementation but also viral genetics could have some influence on immune reconstitution and treatment outcomes, as in our cohort, the patients infected with the B subtype with DRMs achieved higher CD4 count and CD4:CD8 ratio after effective ART implementation. This possible association requires further, direct evaluation. In general, our results align with those of previous studies, which revealed that differences in HIV-1 subtypes A and B did not affect immune reconstitution in effectively treated PWH [42,46].

### 3.3. DRM-Dependent Analysis

#### 3.3.1. DRMs Differences

Studies of the resistance patterns that emerge in patients receiving ART indicate that the presence of polymorphisms before ART initiation may create conditions that facilitate the emergence of specific selection pathways for secondary resistance. Some important differences have been demonstrated between polymorphic and nonpolymorphic DRMs [29,47,48]. Genotypic changes alone do not confer consistently lower treatment susceptibility when viral strains are subjected to phenotypic testing. The magnitude of the reduction in treatment susceptibility conferred by DRMs varies greatly and is modulated by the genetic context of the HIV sequence in which the DRM occurs. Although mutations result in a spectrum of degrees of resistance, they are arbitrarily designated major or accessory mutations. Those defined as major tend to occur earlier in ART failure and confer greater reductions in treatment susceptibility. Conversely, accessory mutations conferring some incremental resistance may occur, as well as polymorphisms, in wild-type viruses and, in some cases, do not reduce treatment susceptibility but may restore replication fitness to viruses with DRMs that impair their fitness. In general, accessory mutations may not be associated with clinical resistance in HIV-1, but in the presence of other mutations, they may contribute to a reduced virological response to a drug, especially when they accumulate. However, polymorphisms associated with impaired ART responses that occur in otherwise wild-type viruses should not be used in epidemiologic analyses to identify transmitted HIV-1 drug resistance [27].

We detected DRMs in 57% of all patients, with the most frequent present mutations for PI (46%). The DRMs for NRTI and NNRTI occurred at rates of 11% and 10%, respectively. Besides A71T, which we detected more frequently in the HIV-1 B subtype, no significant differences between subtypes A and B and the presence of specific DRMs were identified. In the analysis, which included the presence of major and accessory transmitted HIV-1 DRMs together, no drug-specific DRM differences in immune restoration were observed (Table 4).

#### 3.3.2. Possible DRMs-Dependent Mechanisms of Immune Restoration

Persistent inflammatory activation due to HIV infection and coinfections can also cause inadequate immune reconstitution, and the molecular basis of this phenomenon is not fully understood [6,7,8,9]. Despite the effectiveness of ART, HIV remains persistent in the reservoirs, with continuing replication and, thus, chronic immune stimulation and inflammation [10,11,12]. Moreover, some PWH who are virally suppressed on ART occasionally exhibit transiently detectable HIV viral load—blips [49,50]. Suzuki et al. demonstrated that viral blips can reflect increased transcriptional activity from the reservoir and contribute to the reservoir over time. Therefore, residual HIV viremia might lead to systemic immune activation and chronic inflammation, which affects immune reconstitution [51]. In addition, the prevalence of clonal hematopoiesis is higher in PWH with lower CD4 counts and residual HIV transcriptional activity, as well as in virologically suppressed individuals [52]. Furthermore, highly sensitive C-reactive protein (hs-CRP) and Interleukin 6 (IL-6), the most common markers of inflammation, occur at higher concentrations in PWH compared to the general population [7,53]. As DRMs could increase replication in reservoirs (e.g., influence viral replication fitness or increase residual HIV transcriptional activity), they could be a source of immune activation, inflammation, and insufficient CD4 cell restoration in virologically suppressed patients. This issue requires further direct research, as some other viral features have been shown to influence the course of the disease despite effective ART [16,37]. However, in African children who experience viral blips, no significant effect on immune reconstitution has been proven [54].

On the other hand, CD4 depletion and consequent increase in CD4 T cell counts after ART implementation can lead to IRIS [13]. Additionally, excessive immune response to pathogens, invigorated by functional impairments among regulatory T cells, may further induce proinflammatory activation, the possible risk factor of CD4 depletion [6,14,15]. This mechanism could fill the gap between IRIS and DRMs in HIV, but the significance of this association needs to be investigated in targeted clinical trials. In our study in patients with both known risk factors for IRIS, low CD4 T cell counts at the time of diagnosis, and effective ART implementation, we have not observed a coincidence between the HIV subtype or the presence of DRMs with the worse immune reconstitution.

We did not observe that the presence of DRMs was associated with worse immunological restoration, including in the subgroups with HIV drug class mutations. Likewise, we did not detect significant differences in CD4 counts or CD4:CD8 ratio normalization between subgroups with or without DRMs in most cases. Only patients with HIV-1 subtype B and DRMs achieved higher CD4 counts than those with subtype B without DRMs (*p* = 0.013), which was discussed in Section 3.2.3. The analysis revealed that in the subgroup with DRMs, patients were younger (median age 36 vs 41 years, *p* = 0.001; Table 1) and more likely to be male (*p* = 0.003), which corresponds with epidemiological data [31,32,33,39,40]. However, data from Ahn et al. revealed that age >50 years is a risk factor for INRs [55], and in our cohorts, patients were younger in both subgroups, which should not have influenced the results.

### 3.4. Significance and Limitations of This Study

As the frequency of the presence of transmitted DRMs in ART-naïve PWH increases, surveillance should be particularly emphasized. In addition, the reasons for patients’ insufficient immunological response after efficient ART have not been fully elucidated. We did not observe poorer immune reconstitution in patients with a specific subtype (A or B) or the presence of primary DRMs. Effective and tailored ART remains the most important factor for the management of HIV infection.

This study presents an assessment of DRMs for NRTIs, NNRTIs, and PIs, not for the InSTI group of ART. The limited data reflect two factors: the time of recruitment (during the SARS-CoV-2 pandemic), which limited the availability of molecular diagnostics, and the lack of InSTI resistance testing in standard available tests from 2020 to 2022 in recruiting centers.

Moreover, sequencing data analysis consistently points to the lack of current knowledge about the associations between new mutations and drug resistance, the occurrence of relevant mutations outside regions targeted by routine resistance assays, and the risk of the presence of drug-resistant minorities in the HIV population.

Additionally, as CD4 restoration in PWH is complex and multifactorial, and no direct connection between DRMs and this process has been elucidated, a lack of this knowledge is another limitation of our study. Nevertheless, we would like to examine whether inadequate CD4 restoration and the presence of DRM coincide in treatment-naïve PWH to explore a possible connection. Some viral characteristics, such as the HIV subtype, can influence this process and the course of the infection, even in effectively treated individuals, due to the virus–host interaction [16,37]. We decided to search for another viral feature that could influence immune response. Furthermore, the mechanisms of the presence of DRM and their influence on CD4 restoration should be examined pathophysiologically. Possible explanations for the observed phenomena include ongoing replication in reservoirs of HIV strains with higher viral fitness due to the presence of primary DRMs, which could promote inflammatory activity, immune stimulation, and CD4 depletion. The data on this topic are lacking, indicating a need to examine it in the future.

## 4. Materials and Methods

### 4.1. Study Design

We conducted a multicenter, observational, cross-sectional study. Patient data were extracted from a 5-year period spanning from 2020 to 2024. The recruitment sites were four clinical centers in Poland (Warsaw, Ostróda, Lublin, and Rzeszów). There were no differences in the profiles of the clinical centers (each consists of an outpatient clinic and hospital department). The inclusion criteria were HIV-1 infection, no history of ART exposure (ART-naïve patients), and aged 18 years or older. The exclusion criteria were a lack of virological suppression after 6 months of treatment or during the observation period, discontinuation or nonadherence to treatment, and the presence of any hematological malignancies, chemotherapy, and/or iatrogenic immunosuppression. This study was registered on clinicaltrials.gov (NCT06044792) on 3 September 2023.

### 4.2. Clinical Assessment

We collected epidemiological data, such as age, sex, diagnosis of recent HIV infection and the presence of AIDS-defining diseases, and the implementation of the ART regimen. According to the latest European AIDS Clinical Society (EACS) “Guidelines for the management of people living with HIV in Europe” and the European Centre for Disease Prevention and Control (ECDC) recommendations, AIDS was defined as the presence of any illness from AIDS-defining condition list provided by CDC and recent HIV infection was defined as diagnosed up to 6 months after infection: on the basis of seroconversion or diagnosis of acute HIV infection or individuals’ medical history [56,57,58]. We assessed laboratory findings regarding HIV viral load at baseline and every 6 to 12 months after ART initiation, lymphocyte CD4 count, and the CD4:CD8 ratio at the time of diagnosis and 6, 12, 24, 36, and 48 months after ART initiation, in accordance to the EACS guidelines for the management of virologically suppressed PWH [56]. The INR was defined as an individual on effective ART who did not manage to achieve a minimum CD4 cell count ≥500 cells/µL and a CD4:CD8 ratio ≥ 1 [3,34] after treatment implementation. In all patients, just after HIV infection diagnosis and before ART initiation (maximum break period of 7 days), the presence of HIV-1 DRMs to NRTIs, NNRTIs, and PIs was evaluated. The drug resistance was assessed in accordance with the Stanford University HIV Drug Resistance Database [25].

### 4.3. Molecular Investigation

HIV-1 drug resistance genotyping of the HIV-1 pol region, including protease (PR) and reverse transcriptase (RT) coding genes, was performed using the DeepChek HIV-1 PR/RT Drug Resistance Assay (ABL-Advanced Biological Laboratories Diagnostics S.A., Luxembourg), following the manufacturer’s instructions. Template HIV-1 Ribonucleic acid (RNA) was manually extracted from plasma samples collected between 2020 and 2024 from HIV-1-positive patients with viral loads exceeding 1000 copies/mL (the test cutoff). The HIV-1 RNA purification was conducted using the QIAamp Viral RNA Mini Kit (Qiagen, Hilden, Germany). Amplification of HIV-1 pol region fragments was achieved via reverse transcription preceding polymerase chain reaction (RT-PCR) followed by a nested PCR step to enhance sensitivity and specificity. The targeted regions included the protease gene (codons 1 to 99) and codons 1 to 320 of the reverse transcriptase (RT) gene. The obtained amplicon quality and purity were assessed by the visual inspection of the expected molecular sizes of nested PCR products as follows: 937 bp for reverse transcriptase and 520 bp for protease, observed as a result of gel electrophoresis. Nested PCR products were enzymatically purified using an Exo-SAP mix (Exonuclease I and Shrimp Alkaline Phosphatase). The purified products were diluted as necessary, fluorescently labeled with appropriate terminators, and sequenced using an ABI Prism 3500 Genetic Analyzer (Applied Biosystems, Waltham, MA, USA) and the Data Collection Software v4. The sequencing raw data were uploaded to the ViroScore v.3 DeepChek-HIV IVD software (ABL Diagnostics S.A., Luxembourg). Quality control was performed to ensure a required quality threshold of sequence chromatograms was assembled. The software was used to analyze the obtained HIV-1 sequence bases on an updated database of HIV drug resistance-associated mutations and their latest interpretations rules. The assay was validated for HIV-1 subtype B but is also capable of amplifying other subtypes and recombinant forms, such as CRF02_AG. Drug resistance-associated mutations were identified for each sample. Quality-checked pol sequences were also submitted to the Stanford University HIV Drug Resistance Database (https://hivdb.stanford.edu/hivdb/by-sequences/, accessed 22 January 2025) for comparative analysis and interpretation of the genetic variants detected [25]. Subtyping, the identification of circular recombinant forms (CRFs), and drug resistance mutation analysis were further enhanced using online tools provided by the Stanford University HIV Drug Resistance Database and the REGA HIV-1&2 Subtyping Tool. Nucleotide alignments were performed using the MEGA v.12 software package, followed by manual editing.

HIV-1 viral load in plasma samples was quantified using the Cobas 6800/5800 HIV-1 Test (Roche, Rotkreuz, Switzerland). This assay targets two distinct regions of the HIV-1 genome (gag and LTR). The analytical performance parameters were as follows: sensitivity: 13.2 copies/mL, linear range: 20 copies/mL to 1.0 × 10⁷ copies/mL, specificity: 100%.

Immunophenotyping, including the measurement of the absolute count of T lymphocyte CD4+ and CD8+ subsets, was determined using the flow cytometry method with the application of TriTEST fluorescence-conjugated monoclonal antibodies (BD Biosciences, Macquarie Park NSW, Australia). The data were acquired and analyzed with a multichannel dual-laser BD FACSCalibur analyzer using the BD MultiSET software v3.1.

### 4.4. Statistical Evaluation

The statistical evaluation of the collected data was performed in subgroups of PWH with and without HIV DRMs. The analysis was also performed in the subgroups of patients with different HIV-1 subtype infections. The Shapiro–Wilk test was performed to verify the normality of the analyzed variables’ distributions. Student’s *t*-test or the Mann–Whitney U test was used to evaluate the difference in the mean values of continuous variables, and the χ^2^ test or Fisher’s exact test was performed for categorical variables. A *p*-value of <0.05 indicated statistical significance. Statistical analyses were performed using Python 3.7 software and the Statistica 13.1 program (StatSoft, Kraków, Poland).

A CD4 cell count ≥ 500 cells/µL and a CD4:CD8 ratio ≥ 1 were considered within the normal range according to the CDC’s “Guidelines for Performing CD4+ T cell Determinations in Persons with Human Immunodeficiency Virus Infection” [34]. Patients with CD4 counts ≥ 500 cells/µL and a CD4:CD8 ratio ≥ 1.0 at baseline were included in the examination of CD4 cell count and CD4:CD8 ratio growth and excluded from the analysis of CD4 cell count and CD4:CD8 ratio normalization (obtaining a minimum CD4 cell count ≥ 500 cells/µL and a CD4:CD8 ratio ≥ 1). In addition, we assessed the change in the CD4 T cell count over 4 years of observation. We adopted the parameter ΔCD4 cell count, which describes the difference between the baseline CD4 count and the CD4 count after 4 years of sustained ART, and the ΔCD4:CD8 ratio, which describes the difference between the baseline CD4:CD8 ratio and the ratio after 4 years of ART.

### 4.5. Bioethics

This study received a positive opinion from the Bioethics Committee at the Medical University of Warsaw (AKBE/71/2023) on 6 February 2023. Informed consent was obtained from all the subjects involved in this study. All patients’ data analyzed were fully anonymized. This study followed the principles of the Declaration of Helsinki.

## 5. Conclusions

According to our findings, the presence of primary DRMs in different HIV subtypes is not associated with worse immunological reconstitution in patients receiving effective ART. Therefore, tailored treatment and management strategies should be implemented for patient populations at an increased risk of insufficient immune reconstitution and, thus, an elevated risk of mortality, as effective ART remains the most important component of HIV management.

## Figures and Tables

**Table 1:** Baseline patient characteristics.

Variable	Total (*n* = 109)	Patients with Drug Resistance Mutations (*n* = 62)	Patients with No Drug Resistance Mutations (*n* = 47)	*p*	Patients with HIV Subtype A (*n* = 24)	Patients with HIV Subtype B (*n* = 82)	*p*
Age mean (SD)	38.05 (10.61)	35.65 (10.16)	41.21 (10.47)	**0.001**	40.42 (10.21)	36.88 (10.28)	0.050
Male gender *n* (%)	92 (84.40)	58 (93.55)	34 (72.34)	**0.003**	16 (66.67)	76 (92.68)	**0.004**
Recent HIV infection *n* (%)	12 (11.01)	7 (11.29)	5 (10.64)	0.187	4 (16.67)	8 (0.10)	0.462
AIDS-defining disease *n* (%)	38 (34.86)	20 (32.26)	18 (38.30)	0.123	8 (33.33)	28 (34.15)	1.000
Baseline CD4 mean (SD)	247.64 (244.50)	270.02 (282.19)	218.13 (182.18)	0.325	277.17 (246.79)	244.79 (246.85)	0.306
Baseline CD4:CD8 mean (SD)	0.31 (0.28)	0.33 (0.31)	0.30 (0.24)	0.352	0.41 (0.38)	0.29 (0.24)	0.078

**Table 2:** CD4 cell count and CD4:CD8 recovery in patients with HIV subtypes A and B depending on the presence of HIV drug resistance mutations.

Variable	Subtype A with Mutations (*n* = 9)	Subtype A Without Mutations (*n* = 15)	*p*	Subtype B with Mutations (*n* = 50)	Subtype B Without Mutations (*n* = 32)	*p*
Baseline CD4	290.67 (329.32)	269.07 (194.68)	0.5	276.28 (280.87)	194.25 (174.03)	0.170
CD4 after 36–48 months	527.11 (302.85)	524.47 (281.22)	0.983	591.34 (283.46)	450 (165.84)	**0.013**
ΔCD4	236.44 (186.37)	255.40 (165.80)	0.798	315.06 (259.88)	256.69 (140.41)	0.065
CD4 normalization	1 (16.67)	2 (18.18)	1.000	20 (50.00)	11 (36.67)	0.106
No CD4 normalization	5 (83.33)	9 (81.82)	1.000	20 (50.00)	19 (63.33)	0.106
Baseline CD4:CD8	0.46 (0.54)	0.38 (0.25)	0.406	0.32 (0.25)	0.25 (0.22)	0.104
CD4:CD8 after 36–48 months	0.94 (0.74)	0.96 (0.62)	0.441	0.96 (0.74)	0.75 (0.52)	0.062
ΔCD4:CD8	0.48 (0.69)	0.58 (0.46)	0.085	0.64 (0.64)	0.49 (0.44)	0.067
CD4:CD8 normalization	0 (0.00)	4 (36.36)	0.237	13 (32.50)	6 (20.00)	0.108
No CD4:CD8 normalization	6 (100.00)	7 (63.64)	0.237	27 (67.50)	24 (80.00)	0.108

**Table 3:** CD4 cell count and CD4:CD8 recovery in patients with and without HIV drug resistance mutations depending on the HIV subtype.

Variable	Subtype A with Mutations (*n* = 9)	Subtype B with Mutations (*n* = 50)	*p*	Subtype A Without Mutations (*n* = 15)	Subtype B Without Mutations (*n* = 32)	*p*
Baseline CD4	290.67 (329.32)	276.28 (280.87)	0.479	269.07 (194.68)	194.25 (174.03)	0.120
CD4 after 36–48 months	527.11 (302.85)	591.34 (283.46)	0.538	524.47 (281.22)	450 (165.84)	0.266
ΔCD4	236.44 (186.37)	315.06 (259.88)	0.057	255.40 (165.80)	256.69 (140.41)	0.432
CD4 normalization	1 (16.67)	20 (50.00)	0.198	2 (18.18)	11 (36.67)	0.165
No CD4 normalization	5 (83.33)	20 (50.00)	0.198	9 (81.82)	19 (63.33)	0.165
Baseline CD4:CD8	0.46 (0.54)	0.32 (0.25)	0.336	0.38 (0.25)	0.25 (0.22)	0.058
CD4:CD8 after 36–48 months	0.94 (0.74)	0.96 (0.74)	0.384	0.96 (0.62)	0.75 (0.52)	0.142
ΔCD4:CD8	0.48 (0.69)	0.64 (0.64)	**0.047**	0.58 (0.46)	0.49 (0.44)	0.219
CD4:CD8 normalization	0 (0.00)	13 (32.50)	0.163	4 (36.36)	6 (20.00)	0.709
No CD4:CD8 normalization	6 (100.00)	27 (67.50)	0.163	7 (63.64)	24 (80.00)	0.709

**Table 4:** CD4+ T cell count and CD4+CD8+ T cell recovery depending on HIV drug class resistance mutations.

Variable	NRTI Mutations (*n* = 11)	NNRTI Mutations (*n* = 10)	PI Mutations (*n* = 50)	*p*
Baseline CD4	265.27 (309.33)	261.20 (430.21)	291.86 (295.41)	0.942
CD4 after 36–48 months	602.64 (238.39)	392.70 (209.36)	580.24 (291.39)	0.128
ΔCD4	337.36 (341.06)	131.5 (247.41)	288.38 (251.77)	0.170
CD4 normalization	6 (60.00)	1 (12.50)	17 (44.74)	0.117
No CD4 normalization	4 (40.00)	7 (87.50)	21 (55.26)	0.117
Baseline CD4:CD8	0.31 (0.17)	0.23 (0.18)	0.35 (0.33)	0.514
CD4:CD8 after 36–48 months	0.98 (0.39)	0.65 (0.33)	1.00 (0.79)	0.356
ΔCD4:CD8	0.67 (0.28)	0.42 (0.26)	0.66 (0.70)	0.534
CD4:CD8 normalization	4 (40.00)	1 (12.50)	12 (31.58)	0.503
No CD4:CD8 normalization	6 (60.00)	7 (87.50)	26 (68.42)	0.503

## Data Availability

The patients and test results data used to support the findings of this study are included within the article. Previously reported studies (and datasets) used to support this study are cited at relevant places within the text as references.

## Abbreviations

The following abbreviations are used in this manuscript:

HIV	Human Immunodeficiency Virus
PWH	People with HIV
ART	Antiretroviral therapy
INR	Immunological non-responder
AIDS	Acquired Immunodeficiency Syndrome
IRIS	Immune Reconstitution Inflammatory Syndrome
DRM	Drug resistance mutation
HIV-1	HIV type 1
PI	Protease Inhibitor
NRTI	Nucleoside Reverse Transcriptase Inhibitor
NNRTI	Non-nucleoside Reverse Transcriptase Inhibitor
InSTI	Integrase Strand Transfer Inhibitor
CDC	Centers for Disease Control and Prevention
CRF	Circulating Recombinant Form
URF	Unique Recombinant Form
hs-CRP	highly sensitive C-Reactive Protein
IL-6	Interleukin 6
EACS	European AIDS Clinical Society
ECDC	European Centre for Disease Prevention and Control
PR	Protease
RT	Reverse Transcriptase
RNA	Ribonucleic acid
PCR	Polymerase Chain Reaction

## References

[1] Marcus J.L., Leyden W.A., Alexeeff S.E., Anderson A.N., Hechter R.C., Hu H., Lam J.O., Towner W.J., Yuan Q., Horberg M.A. (2020). Comparison of Overall and Comorbidity-Free Life Expectancy Between Insured Adults with and Without HIV Infection, 2000–2016. JAMA.

[2] Gazzola L., Tincati C., Bellistrì G.M., Monforte A.D., Marchetti G. (2009). The absence of CD4^+^ T cell count recovery despite receipt of virologically suppressive highly active antiretroviral therapy: Clinical risk, immunological gaps, and therapeutic options. Clin. Infect. Dis..

[3] Yang X., Su B., Zhang X., Liu Y., Wu H., Zhang T. (2020). Incomplete immune reconstitution in HIV/AIDS patients on antiretroviral therapy: Challenges of immunological non-responders. J. Leukoc. Biol..

[4] Li C.X., Li Y.Y., He L.P., Kou J., Bai J.S., Liu J., Tian B., Cao L.J., Wang K.H., Kuang Y.Q. (2019). The predictive role of CD4^+^ cell count and CD4/CD8 ratio in immune reconstitution outcome among HIV/AIDS patients receiving antiretroviral therapy: An eight-year observation in China. BMC Immunol..

[5] Wang X., Xiao J., Zhang L., Liu Y., Chen N., Deng M., Song C., Liu T., Zhang Y., Zhao H. (2023). Longitudinal analysis of immune reconstitution and metabolic changes in women living with HIV: A real-world observational study. Chin. Med. J..

[6] So-Armah K., Benjamin L.A., Bloomfield G.S., Feinstein M.J., Hsue P., Njuguna B., Freiberg M.S. (2020). HIV and cardiovascular disease. Lancet HIV.

[7] Borges Á.H., O’Connor J.L., Phillips A.N., Rönsholt F.F., Pett S., Vjecha M.J., French M.A., Lundgren J.D., INSIGHT SMART and ESPRIT Study Groups and the SILCAAT Scientific Committee (2015). Factors Associated With Plasma IL-6 Levels During HIV Infection. J. Infect. Dis..

[8] Guo H., Gao J., Taxman D.J., Ting J.P., Su L. (2014). HIV-1 infection induces interleukin-1β production via TLR8 protein-dependent and NLRP3 inflammasome mechanisms in human monocytes. J. Biol. Chem..

[9] Chinnapaiyan S., Dutta R.K., Nair M., Chand H.S., Rahman I., Unwalla H.J. (2019). TGF-β1 increases viral burden and promotes HIV-1 latency in primary differentiated human bronchial epithelial cells. Sci. Rep..

[10] Wang Y., Lifshitz L., Silverstein N.J., Mintzer E., Luk K., StLouis P., Brehm M.A., Wolfe S.A., Deeks S.G., Luban J. (2023). Transcriptional and chromatin profiling of human blood innate lymphoid cell subsets sheds light on HIV-1 pathogenesis. EMBO J..

[11] Grozdeva R., Ivanov D., Strashimirov D., Kapincheva N., Yordanova R., Mihailova S., Georgieva A., Alexiev I., Grigorova L., Partsuneva A. (2024). Relationship between Modern ART Regimens and Immunosenescence Markers in Patients with Chronic HIV Infection. Viruses.

[12] Vandergeeten C., Fromentin R., Chomont N. (2012). The role of cytokines in the establishment, persistence and eradication of the HIV reservoir. Cytokine Growth Factor Rev..

[13] Thapa S., Shrestha U. (2025). Immune Reconstitution Inflammatory Syndrome. StatPearls.

[14] Meya D.B., Manabe Y.C., Boulware D.R., Janoff E.N. (2016). The immunopathogenesis of cryptococcal immune reconstitution inflammatory syndrome: Understanding a conundrum. Curr. Opin. Infect. Dis..

[15] Martin-Blondel G., Mars L.T., Liblau R.S. (2012). Pathogenesis of the immune reconstitution inflammatory syndrome in HIV-infected patients. Curr. Opin. Infect. Dis..

[16] Parczewski M., Scheibe K., Witak-Jędra M., Pynka M., Aksak-Wąs B., Urbańska A. (2021). Infection with HIV-1 subtype D adversely affects the live expectancy independently of antiretroviral drug use. Infect. Genet. Evol..

[17] Venner C.M., Nankya I., Kyeyune F., Demers K., Kwok C., Chen P.L., Rwambuya S., Munjoma M., Chipato T., Byamugisha J. (2016). Infecting HIV-1 Subtype Predicts Disease Progression in Women of Sub-Saharan Africa. EBioMedicine.

[18] Widiyanti M., Hadi M.I. (2019). Viral and Host Factors are Related to the Progression of HIV Diseases in Mimika, Papua. Open Access Maced. J. Med. Sci..

[19] Alaeus A., Lidman K., Björkman A., Giesecke J., Albert J. (1999). Similar rate of disease progression among individuals infected with HIV-1 genetic subtypes A-D. AIDS.

[20] McPhee E., Grabowski M.K., Gray R.H., Ndyanabo A., Ssekasanvu J., Kigozi G., Makumbi F., Serwadda D., Quinn T.C., Laeyendecker O. (2019). The interaction of HIV set point viral load and subtype on disease progression. AIDS Res. Hum. Retrovir..

[21] Kiguoya M.W., Mann J.K., Chopera D., Gounder K., Lee G.Q., Hunt P.W., Martin J.N., Ball T.B., Kimani J., Brumme Z.L. (2017). Subtype-specific differences in Gag-protease-driven replication capacity are consistent with intersubtype differences in HIV-1 disease progression. J. Virol..

[22] Gupta R.K., Hill A., Sawyer A.W., Cozzi-Lepri A., von Wyl V., Yerly S., Lima V.D., Günthard H.F., Gilks C., Pillay D. (2009). Virological monitoring and resistance to first-line highly active antiretroviral therapy in adults infected with HIV-1 treated under WHO guidelines: A systematic review and meta-analysis. Lancet Infect. Dis..

[23] Wheeler W.H., Ziebell R.A., Zabina H., Pieniazek D., Prejean J., Bodnar U.R., Mahle K.C., Heneine W., Johnson J.A., Hall H.I. (2010). Prevalence of transmitted drug resistance associated mutations and HIV-1 subtypes in new HIV-1 diagnoses, U.S.-2006. AIDS.

[24] Bokharaei-Salim F., Esghaei M., Khanaliha K., Kalantari S., Marjani A., Fakhim A., Keyvani H. (2020). HIV-1 reverse transcriptase and protease mutations for drug-resistance detection among treatment-experienced and naïve HIV-infected individuals. PLoS ONE.

[25] Stanford University HIV Drug Resistance Database. http://hivdb.stanford.edu.

[26] Rhee S.Y., Kassaye S.G., Barrow G., Sundaramurthi J.C., Jordan M.R., Shafer R.W. (2020). HIV-1 transmitted drug resistance surveillance: Shifting trends in study design and prevalence estimates. J. Int. AIDS Soc..

[27] Wensing A.M., Calvez V., Ceccherini-Silberstein F., Charpentier C., Günthard H.F., Paredes R., Shafer R.W., Richman D.D. (2022). 2022 update of the drug resistance mutations in HIV-1. Top. Antivir. Med..

[28] Branda F., Giovanetti M., Sernicola L., Farcomeni S., Ciccozzi M., Borsetti A. (2024). Comprehensive Analysis of HIV-1 Integrase Resistance-Related Mutations in African Countries. Pathogens.

[29] Zhukova A., Dunn D., Gascuel, O., on behalf of the UK HIV Drug Resistance Database & the Collaborative HIV, Anti-HIV Drug Resistance Network (2023). Modeling Drug Resistance Emergence and Transmission in HIV-1 in the UK. Viruses.

[30] Pingarilho M., Pimentel V., Diogo I., Fernandes S., Miranda M., Pineda-Pena A., Libin P., Theys K., Martins M.R.O., Vandamme A.M. (2020). Increasing Prevalence of HIV-1 Transmitted Drug Resistance in Portugal: Implications for First Line Treatment Recommendations. Viruses.

[31] Kirichenko A., Lapovok I., Baryshev P., van de Vijver D.A.M.C., van Kampen J.J.A., Boucher C.A.B., Paraskevis D., Kireev D. (2020). Genetic Features of HIV-1 Integrase Sub-Subtype A6 Predominant in Russia and Predicted Susceptibility to INSTIs. Viruses.

[32] Serwin K., Urbańska A., Scheibe K., Witak-Jędra M., Jankowska M., Hlebowicz M., Bociąga-Jasik M., Kalinowska-Nowak A., Biała M., Ciepłucha H. (2021). Molecular epidemiology and HIV-1 variant evolution in Poland between 2015 and 2019. Sci. Rep..

[33] Beyrer C., Wirtz A.L., O’Hara G., Léon N., Kazatchkine M. (2017). The expanding epidemic of HIV-1 in the Russian Federation. PLoS Med..

[34] CDC (1997). Revised Guidelines for Performing CD4+ T-Cell Determinations in Persons Infected with Human Immunodeficiency Virus (HIV). https://www.cdc.gov/mmwr/preview/mmwrhtml/00045580.htm.

[35] Serrano-Villar S., Deeks S.G. (2015). CD4/CD8 ratio: An emerging biomarker for HIV. Lancet HIV.

[36] Lee S.S., Wong N.S., Wong B.C.K., Wong K.H., Chan K.C.W. (2017). Combining CD4 recovery and CD4: CD8 ratio restoration as an indicator for evaluating the outcome of continued antiretroviral therapy: An observational cohort study. BMJ Open.

[37] Caby F., Guihot A., Lambert-Niclot S., Guiguet M., Boutolleau D., Agher R., Valantin M.A., Tubiana R., Calvez V., Marcelin A.G. (2016). Determinants of a Low CD4/CD8 Ratio in HIV-1-Infected Individuals Despite Long-term Viral Suppression. Clin. Infect. Dis..

[38] Mussini C., Lorenzini P., Cozzi-Lepri A., Lapadula G., Marchetti G., Nicastri E., Cingolani A., Lichtner M., Antinori A., Gori A. (2015). CD4/CD8 ratio normalization and non-AIDS-related events in individuals with HIV who achieve viral load suppression with antiretroviral therapy: An observational cohort study. Lancet HIV.

[39] Hemelaar J., Elangovan R., Yun J., Dickson-Tetteh L., Kirtley S., Gouws-Williams E., Ghys P.D., WHO-UNAIDS Network for HIV Isolation and Characterisation (2020). Global and regional epidemiology of HIV-1 recombinants in 1990–2015: A systematic review and global survey. Lancet HIV.

[40] European Centre for Disease Prevention and Control/WHO Regional Office for Europe (2021). HIV/AIDS Surveillance in Europe 2021-2020 Data. Stockholm: ECDC. https://www.ecdc.europa.eu/en/publications-data/hiv-aids-surveillance-europe-2021-2020-data.

[41] Blassel L., Tostevin A., Villabona-Arenas C.J., Peeters M., Hué S., Gascuel O., UK HIV Drug Resistance Database (2021). Using machine learning and big data to explore the drug resistance landscape in HIV. PLoS Comput. Biol..

[42] Sarabia I., Bosque A. (2019). HIV-1 Latency and Latency Reversal: Does Subtype Matter?. Viruses.

[43] Ceccarelli G., Giovanetti M., Sagnelli C., Ciccozzi A., d’Ettorre G., Angeletti S., Borsetti A., Ciccozzi M. (2021). Human Immunodeficiency Virus Type 2: The Neglected Threat. Pathogens.

[44] Nowicka-Sans B., Gong Y.F., McAuliffe B., Dicker I., Ho H.T., Zhou N., Eggers B., Lin P.F., Ray N., Wind-Rotolo M. (2021). In vitro antiviral characteristics of HIV-1 attachment inhibitor BMS-626529, the active component of the prodrug BMS-663068. Antimicrob. Agents Chemother..

[45] Li K., Chen H., Li J., Feng Y., Lan G., Liang S., Liu M., Rashid A., Xing H., Shen Z. (2022). Immune reconstruction effectiveness of combination antiretroviral therapy for HIV-1 CRF01_AE cluster 1 and 2 infected individuals. Emerg. Microbes Infect..

[46] Fokam J., Santoro M.M., Takou D., Njom-Nlend A.E., Ndombo P.K., Kamgaing N., Kamta C., Essiane A., Sosso S.M., Ndjolo A. (2019). Evaluation of treatment response, drug resistance and HIV-1 variability among adolescents on first- and second-line antiretroviral therapy: A study protocol for a prospective observational study in the centre region of Cameroon (EDCTP READY-study). BMC Pediatr..

[47] Bhargava M., Cajas J.M., Wainberg M.A., Klein M.B., Pant Pai N. (2014). Do HIV-1 non-B subtypes differentially impact resistance mutations and clinical disease progression in treated populations? Evidence from a systematic review. J. Int. AIDS Soc..

[48] Tamalet C., Tissot-Dupont H., Motte A., Tourrès C., Dhiver C., Ravaux I., Poizot-Martin I., Dieng T., Tomei C., Bregigeon S. (2018). Emergence of uncommon HIV-1 non-B subtypes and circulating recombinant forms and trends in transmission of antiretroviral drug resistance in patients with primary infection during the 2013-2015 period in Marseille, Southeastern France. J. Med. Virol..

[49] Oomen P.G.A., Dijkstra S., Hofstra L.M., Nijhuis M.M., Verbon A., Mudrikova T., Wensing A.M.J., Hoepelman A.I.M., Van Welzen B.J. (2023). Integrated analysis of viral blips, residual viremia, and associated factors in people with HIV: Results from a retrospective cohort study. J. Med. Virol..

[50] Han W.M., Broom J., Bopage R., Templeton D.J., Edmiston N., Petoumenos K., Australian HIV Observational Database (2024). Investigating rates and predictors of viral blips, low-level viraemia and virological failure in the Australian HIV observational database. Trop. Med. Int. Health.

[51] Suzuki K., Levert A., Yeung J., Starr M., Cameron J., Williams R., Rismanto N., Stark T., Druery D., Prasad S. (2021). HIV-1 viral blips are associated with repeated and increasingly high levels of cell-associated HIV-1 RNA transcriptional activity. AIDS.

[52] van der Heijden W.A., van Deuren R.C., van de Wijer L., van den Munckhof I.C.L., Steehouwer M., Riksen N.P., Netea M.G., de Mast Q., Vandekerckhove L., de Over R.M. (2022). Clonal Hematopoiesis Is Associated With Low CD4 Nadir and Increased Residual HIV Transcriptional Activity in Virally Suppressed Individuals With HIV. J. Infect. Dis..

[53] Sharif S., Van der Graaf Y., Cramer M.J., Kapelle L.J., de Borst G.J., Visseren F.L.J., Westerink J., SMART study group (2021). Low-grade inflammation as a risk factor for cardiovascular events and all-cause mortality in patients with type 2 diabetes. Cardiovasc. Diabetol..

[54] Prendergast A.J., Szubert A.J., Pimundu G., Berejena C., Pala P., Shonhai A., Hunter P., Arrigoni F.I.F., Musiime V., Bwakura-Dangarembizi M. (2021). The impact of viraemia on inflammatory biomarkers and CD4^+^ cell subpopulations in HIV-infected children in sub-Saharan Africa. AIDS.

[55] Ahn M.Y., Jiamsakul A., Khusuwan S., Khol V., Pham T.T., Chaiwarith R., Avihingsanon A., Kumarasamy N., Wong W.W., Kiertiburanakul S. (2019). IeDEA Asia-Pacific. The influence of age-associated comorbidities on responses to combination antiretroviral therapy in older people living with HIV. J. Int. AIDS Soc..

[56] EACS 2024 Guidelines for the Management of People Living with HIV in Europe. https://eacs.sanfordguide.com/.

[57] European Centre for Disease Prevention and Control HIV Infection and AIDS. https://www.ecdc.europa.eu/en/hiv-infection-and-aids.

[58] MMWR Appendix A—AIDS-Defining Conditions. https://www.cdc.gov/mmwr/preview/mmwrhtml/rr5710a2.htm.

